# Measurement of Rock Joint Surfaces by Using Smartphone Structure from Motion (SfM) Photogrammetry

**DOI:** 10.3390/s21030922

**Published:** 2021-01-30

**Authors:** Pengju An, Kun Fang, Qiangqiang Jiang, Haihua Zhang, Yi Zhang

**Affiliations:** 1 Faculty of Engineering, China University of Geosciences, Wuhan 430074, China; apj@cug.edu.cn; 2 Wuhan Design and Research Institute Co., Ltd., China Coal Technology & Engineering Group Corp, Wuhan 430064, China; jiangqiangqiang@zmwhy.com.cn; 3 Technical Research & Development Institute, Kumagai Gumi Co., Ltd., Ibaraki 3002651, Japan; haihua.zhang@ku.kumagaigumi.co.jp; 4 Department of Civil Engineering, Tsinghua University, Beijing 100084, China; zhang-yi@tsinghua.edu.cn

**Keywords:** rock joint, structure from motion (SfM), photogrammetry, smartphone

## Abstract

The measurement of rock joint surfaces is essential for the estimation of the shear strength of the rock discontinuities in rock engineering. Commonly used techniques for the acquisition of the morphology of the surfaces, such as profilometers and laser scanners, either have low accuracy or high cost. Therefore, a high-speed, low-cost, and high-accuracy method for obtaining the topography of the joint surfaces is necessary. In this paper, a smartphone structure from motion (SfM) photogrammetric solution for measuring rock joint surfaces is presented and evaluated. Image datasets of two rock joint specimens were taken under two different modes by using an iPhone 6s, a Pixel 2, and a T329t and subsequently processed through SfM-based software to obtain 3D models. The technique for measuring rock joint surfaces was evaluated using the root mean square error (RMSE) of the cloud-to-cloud distance and the mean error of the joint roughness coefficient (JRC). The results show that the RMSEs by using the iPhone 6s and Pixel 2 are both less than 0.08 mm. The mean errors of the JRC are −7.54 and −5.27% with point intervals of 0.25 and 1.0 mm, respectively. The smartphone SfM photogrammetric method has comparable accuracy to a 3D laser scanner approach for reconstructing laboratory-sized rock joint surfaces, and it has the potential to become a popular method for measuring rock joint surfaces.

## 1. Introduction

Rock joints play a crucial role in engineering projects and applications related to rock masses, since they significantly affect the mechanical and hydrological properties of rock masses, such as their strength and permeability [1,2]. Generally, the surfaces of rock joints are rough rather than smooth. In addition to other factors, including lithology, confining pressure, water content, etc., the joint roughness is a dominant parameter in the shear strength of rock joints. Therefore, the quantification of rock joint roughness is essential for assessing the engineering stability of rock masses. As a parameter for quantification in practice, the rock joint roughness coefficient (JRC), estimated initially by comparing the morphological parameters of standard joint profiles [3], is widely used to evaluate the peak shear strength of rock discontinuities [4]. However, one of the shortcomings is that Barton’s JRC method only focuses on the ten two dimensional (2D) profiles via visual comparison [5].

The subsequent studies on JRC, therefore, shift gradually from visual comparison with traditional Barton’s profiles to the measurement of 2D profiles or 3D surfaces by using different techniques. Early studies on the JRC primarily focused on measuring the characteristics of 2D rock joint profiles by using profilometers [6,7]. Later, 3D laser scanners were gradually used to measure the surface topography of rock joints in the laboratory with high resolution and accuracy [8,9,10]. However, expensive equipment is needed to obtain 3D models of rock joint surfaces, which restricts the wide use of this technology in the measurement of rock joint surfaces. Therefore, a low-cost approach for acquiring the 3D data of rock joint surfaces is in high demand.

With the development of computer vision theory and automatic feature-matching algorithms, a 3D reconstruction approach called structure from motion (SfM) has been introduced into photogrammetry [11,12]. Compared with other traditional close-range photogrammetry methods, the SfM algorithm has many advantages, such as high efficiency, flexible workflows, and no expert supervision. One characteristic feature of the algorithm is that the 3D positions and shooting angles of each camera and the object space coordinates can be automatically estimated by detecting and matching the featured points from a set of overlapping images [13,14]. Based on the SfM algorithm, some free tools (e.g., Visual SFM, OpenMVG, and ARC 3D Webservice) and commercial software (e.g., Agisoft Metashape, Pix4Dmapper, and PhotoModeler) have been developed to process various types of images taken from different camera sensors. Among these software packages, Agisoft Metashape, formerly known as Agisoft Photoscan, is a simple and popular solution for photogrammetric applications [15,16]. Through the software, inexperienced users can obtain accurate 3D models in only a few steps.

With the innovation of phone technology, smartphones have the potential to be applied in many fields. Most current smartphones are equipped with high image resolution cameras and powerful central processing units, which can enhance the quality of the captured images. Additionally, portability and accessibility are characteristic features of smartphones. Thus, many researchers have tested the performance of smartphone cameras for 3D reconstruction. Akca and Gruen [17] studied the radiometric accuracy of smartphone cameras, including a Sony Ericsson K750i and a Nokia N93, and compared the results with those of single-lens reflex cameras. The results showed that properly calibrated smartphone cameras can be used for photogrammetric tasks with a required accuracy of 1:10,000. The smartphone SfM photogrammetric method has been tested in many fields including flexible structural monitoring [18], coastal monitoring [19,20,21], auxiliary medical examinations [22,23], alpine alluvial fan surveys [24], and soil water erosion [25]. Although the smartphone SfM photogrammetric method has been successfully used in these applications and has produced high-resolution results, to the best of the authors’ knowledge, this approach has rarely been used for mapping the topography of rock joint surfaces in geotechnical laboratories. In addition, the effects of smartphone camera, shooting mode, and rock joint type on the accuracy of the method are not investigated systematically. Therefore, evaluating the accuracy of this approach is useful and necessary for measuring rock joint surfaces.

In this study, a low-cost solution for measuring the topography of laboratory-sized rock joint surfaces is presented. Three main factors affecting the implementation of the method are considered: smartphone camera quality, specimen texture, and shooting strategy. The accuracy of this solution was assessed in two aspects consisting of the cloud-to-cloud distance of 3D models and the JRC of 2D profiles by comparing the data obtained from a handheld 3D laser scanner (HLS). Finally, some advantages and limitations of using this technology are discussed.

## 2. Materials and Methods

**Figure 1:**
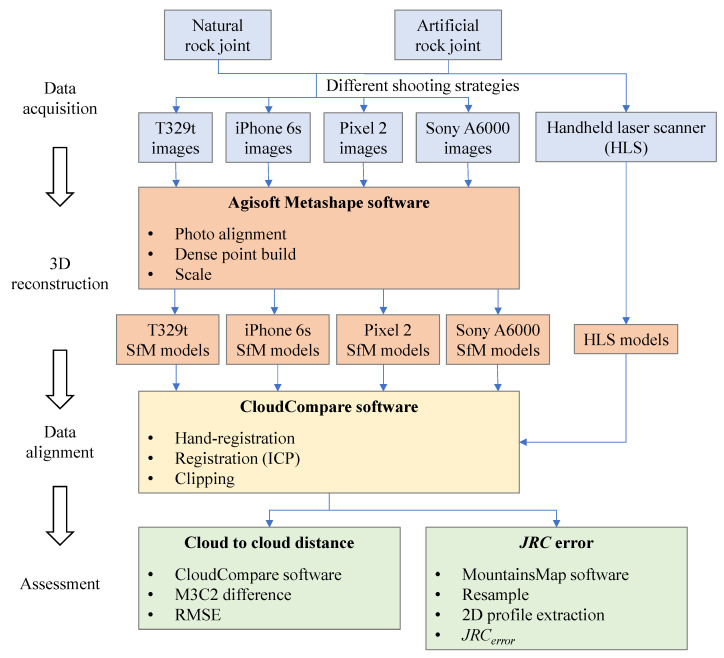
Overall workflow of the methodology.

The overall workflow adopted in the reconstruction of the rock joint surfaces through smartphone SfM photogrammetry and assessment of the method is presented in Figure 1. Four main steps including data acquisition, 3D reconstruction, coregistration, and assessment were performed to obtain the 3D models of rock joint surfaces and evaluate the accuracy of the method. In the evaluation, three factors, including the smartphone camera quality, specimen texture, and shooting strategy, were tested.

### 2.1. Data Acquisition

#### 2.1.1. Image Acquisition

Three widely used smartphones, namely, the HTC T329t (HTC, New Taipei City, Taiwan), Apple iPhone 6s (Apple, Cupertino, CA, USA) and Google Pixel 2 (Mountain View, CA, USA), were selected as the test equipment to capture images and to evaluate the effect of the smartphone camera quality on 3D reconstruction. The T329t equipped with a 5 MP (2592 × 1552) camera is a phone with a relatively low shooting capability released in 2012. The other two smartphones, both with 12 MP (4032 × 3024) cameras, were released in 2015 and 2017. Although the three smartphones have been released for three to eight years, the performances of the equipped cameras comply with the basic requirements of this study based on some preliminary tests. Moreover, one advantage is that the three phones can be acquired at low prices in second-hand markets to conduct the tests. To further assess the smartphone camera quality in SfM photogrammetry, a Sony A6000 (Sony, Minato City, Tokyo, Japan) high-end camera with a SELP1650 (Sony, Minato City, Tokyo, Japan) zoom lens was used. All detailed parameters of the three smartphone cameras and the high-end camera are summarized in Table 1.

**Figure 2:**
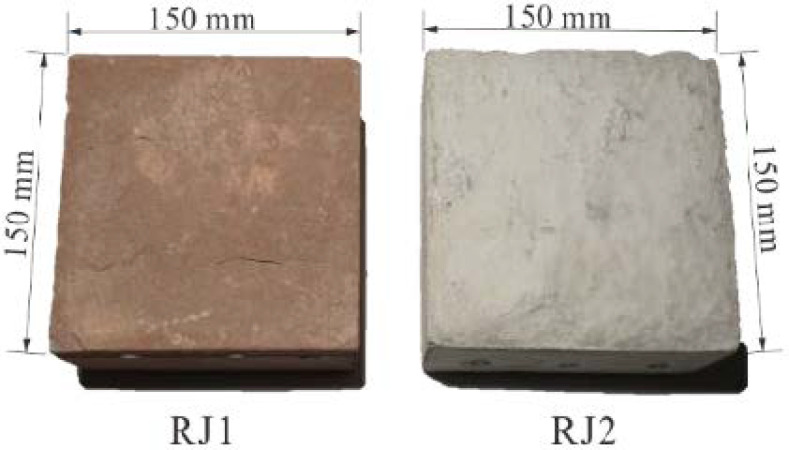
Two rock joint specimens used for 3D reconstruction, RJ1 is a natural red sandstone rock joint specimen, RJ2 is an artificial rock joint specimen.

Naturally, the size and texture of rock joints affect the reconstruction quality. According to the International Society for Rock Mechanics [26], the size and shape of the specimen for the laboratory determination of the shear strength of the rock joint are recommended to have a regular cross section, and the width of the test plane should be at least 48 mm. According to the commonly used direct shear apparatus, square-shaped specimens with side lengths of 100 or 150 mm were mainly used [27,28]. For the texture of the rock joint surface, textures with dull colors and repeating or symmetrical patterns may cause the resultant 3D model to be incomplete, decrease the accuracy of the model, and even lead to 3D reconstruction failure. Usually, natural rock joint surfaces tend to have rich textures, while artificial joint surfaces have relatively homogeneous textures. Considering the size and textural characteristics of the rock joint mentioned above, we chose a natural red sandstone (RJ1) and an artificial rock joint (RJ2), as shown in Figure 2. The sizes of the two specimens are both 150 × 150 mm. The natural rock joint was acquired by splitting it from a rock mass, while the artificial specimen with a smooth and monochromatic surface was built using cement, sand, and water at a ratio of 1:1:0.45 by weight.

In addition to the camera quality and the specimen texture, the shooting strategy consisting of the shooting mode and shooting angle for 3D reconstruction are also evaluated. Regarding the shooting mode, rotating the rock joint on a turntable with a fixed smartphone and moving the smartphone around the rock joint, namely, the fixed smartphone capture (FSC) mode and the moving smartphone capture (MSC) mode, were tested. Under the FSC mode, the distance of the smartphone camera from the object and the illumination can be controlled precisely, and camera shake can be avoided during shooting. The MSC mode is a quick and straightforward approach without special settings. Additionally, different shooting angles from the smartphone to the surface of the rock joint are investigated under the FSC mode.

**Figure 3:**
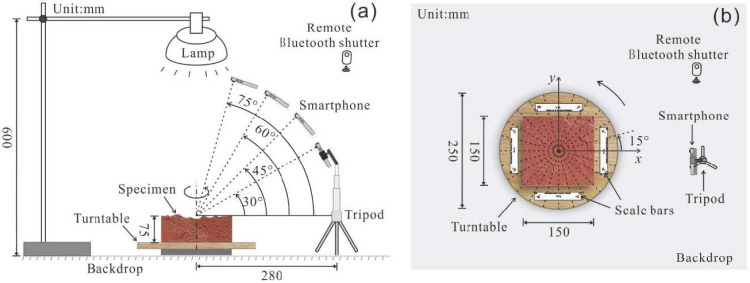
Schematic diagram of fixed smartphone capturing (FSC) mode (**a**) side view, (**b**) top view.

In the setup of the FSC mode, the specimen was placed at the center of the turntable (see Figure 3a). A featureless white polyvinyl chloride (PVC) sheet under the turntable was used as the backdrop to avoid the mismatching of feature points in the Metashape software (Agisoft, Saint Petersburg, Russian Federation). The smartphone was fixed on a tripod and triggered by a remote Bluetooth shutter to prevent camera shake. The distance between the smartphone and specimen was set at 30 cm to enable the entire specimen to be encompassed in the frame. After preparing the specimen and setting up the smartphone, the turntable was rotated every 15° accompanied by a picture being shot (see Figure 3b). Thus, a total of 24 overlapping images, considered as a dataset, were captured for a rock joint surface. Additionally, four different shooting angles, including 30, 45, 60, and 75°, were tested, as shown in Figure 3a. For the MSC mode, each specimen was placed on the ground and photographed manually around the specimen. Due to the handheld shooting, the distance from the camera to the specimen was 25–40 mm, and the shooting angle was 50–70°. Pictures were taken of the rock joint surfaces after moving the smartphones every 12–18°. Finally, 24 images under the MSC mode were also obtained for each specimen.

**Figure 4:**
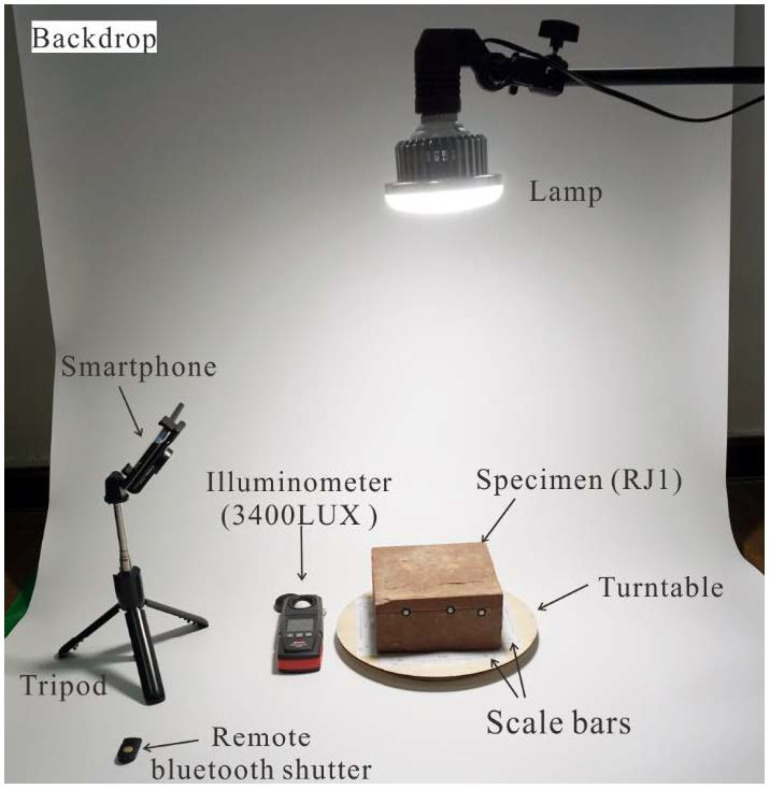
The setup of image acquisition under the FSC mode.

During the image acquisition process, an ordinary table lamp with a semitranslucent lampshade was used to build a diffuse light condition above the specimen, as shown in Figure 4. The illuminance on the specimen surface was 3400 lux, as measured by a PP720 digital lux meter produced by SanLiang company in Guangdong, China. In addition, four designed scale bars with a distance of 10 cm, considered coded markers, were prepared in black-and-white color to offer a better image alignment in the Metashape software, as shown in Figure 4.

#### 2.1.2. Data Acquisition from a 3D Laser Scanner

**Figure 5:**
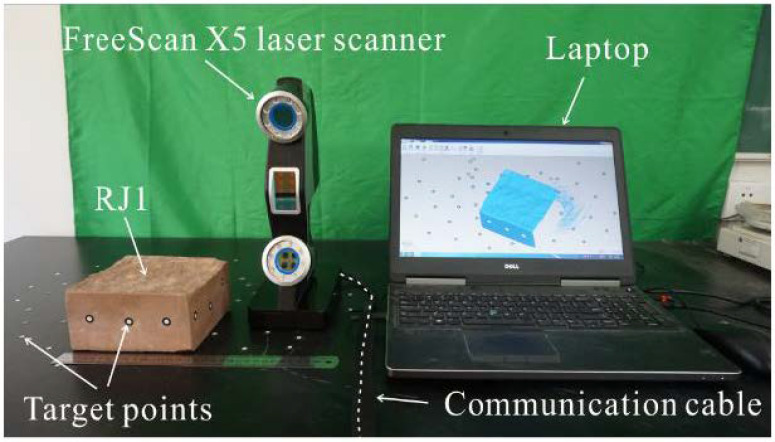
Handheld laser scanner of FreeScan X5.

The benchmark data were acquired by using a 3D HLS FreeScan X5 manufactured by the Shining company in Beijing, China (see Figure 5). This ultra-portable laser scanner is suitable for specimens with complex shapes, especially for rock joints with rough surfaces. The maximum resolution of the scanner is 0.1 mm at a distance of 30 cm. The scanning speed of the laser scanner can reach 350,000 dots/s, and all the point information can be synced to a laptop through a USB cable simultaneously. Consequently, the 3D model of an object can be reconstructed in near real time. The detailed parameters of the HLS are given in Table 2.

To estimate the JRC of rock joints, the sampling interval is usually 0.1–2 mm [29]. As a benchmark, the scanning interval was set at 0.2 mm to obtain a high-density point cloud. Several target points with a diameter of 3 mm were fixed around the specimen to increase the alignment accuracy (see Figure 5). During the scanning process, continuous scanning from different angles and positions was conducted to generate a complete specimen point cloud. Finally, the point clouds of the rock joint surfaces through the HLS, named HLS models, were obtained and used as a benchmark in the assessment.

### 2.2. Three-Dimensional Model Reconstruction

**Figure 6:**
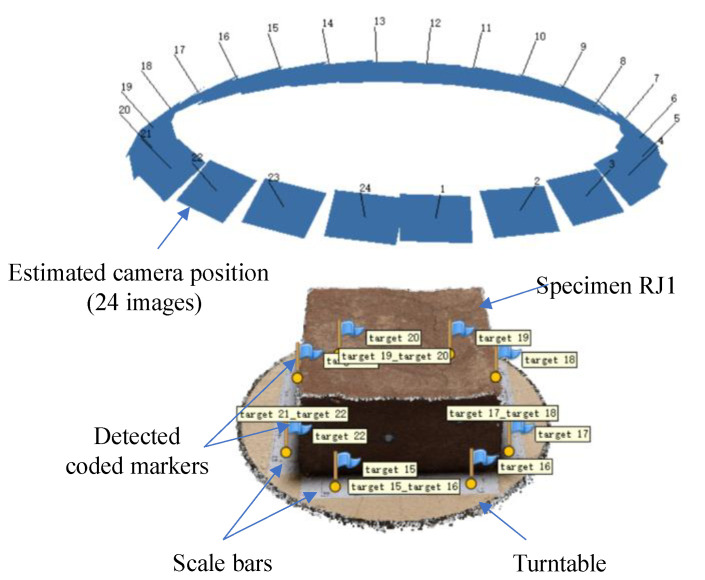
A 3D model of RJ1 reconstructed by Metashape software.

After acquiring the image datasets, the 3D models of the rock joint surfaces were reconstructed by using the Metashape software. The 3D models of rock joint surfaces are mainly created by three stages: aligning photos, building dense point clouds, and scaling. In the process of aligning photos, the feature points from overlapping images were automatically detected and matched. Here, the setting of high accuracy for aligning was selected in the software. Then, the position and calibration parameters of each camera were obtained. Self-calibration is an effective method to estimate the camera’s internal parameters in SfM photogrammetry and has been widely used [30]. Since the camera calibration parameters were determined in the process of bundle adjustment, users do not need any additional operations in the workflow. Table 3 shows the self-calibration results of four different cameras. The calibration parameters include focal length (*f*), principal point coordinates (*cx*, *cy*), radial distortion coefficients (*k*1, *k*2, *k*3), and tangential distortion coefficients (*p*1, *p*2). The calculated parameters have a variability for the same camera with different image datasets mentioned in Section 2.1, which is closely related to the noise in complementary metal oxide semiconductor (CMOS) image sensors [31]. Based on the image datasets and the acquired camera parameters, dense point clouds with random dimensions were generated. The scale bars were subsequently used to scale the real size of the dense point clouds. Finally, the point clouds of the rock joint surfaces at actual size, called SfM models, were generated, as shown in Figure 6.

### 2.3. Data Alignment

Since the acquired SfM and HLS models were under different coordinate systems, the two models cannot be directly compared. Thus, the coregistration of the two models was conducted and prepared for the next assessment. In the coregistration processing, since the HLS model was set as the benchmark, only the SfM model was rotated and translated by using the CloudCompare software. The rough rotation and translation of the SfM model were first manually operated to match the HLS model. To further improve the registration accuracy, an iterative closest point algorithm was adopted to reduce coregistration errors [32]. The algorithm iteratively calculates a transformation matrix to minimize the spatial distance between the two different point clouds. Here, the random sampling points and root mean square difference were set to 100,000 and 1 × 10^−5^ m, respectively, in the software. Finally, the optimized transformation matrix was used to fit the SfM model to the HLS model, which ensures that the two point clouds are in the same coordinate system. In addition, in order to avoid the effect of noisy points at the edge of the SfM model in the comparison, the coregistered SfM and HLS models were manually clipped to a rectangular area that was smaller than the real size of the rock joint surface. The length of the rectangle was 142.1–148.7 mm.

### 2.4. Assessment of the Accuracy of Smartphone SfM Photogrammetry

In this study, two parameters, including the cloud-to-cloud distance and JRC error, were used to form 3D and 2D quantitative assessments of the SfM model of the rock joints, respectively. By considering HLS models as the benchmark, the cloud-to-cloud distance between the SfM and HLS models can reflect the accuracy of the 3D SfM models. To further evaluate the accuracy of the 2D cross sections of the SfM models, the JRCs of the 2D profiles extracted from the SfM and HLS models were compared.

#### 2.4.1. Cloud-to-Cloud Distance

The cloud-to-cloud distance of the two models is calculated by using the multiscale model to model cloud comparison (M3C2) algorithm in CloudCompare. Unlike calculations of the nearest neighbor distance in traditional algorithms, the M3C2 algorithm operates by computing the distance along a local normal direction estimated from the local surface roughness, which can effectively reduce the effects of point position uncertainty and registration error.

**Figure 7:**
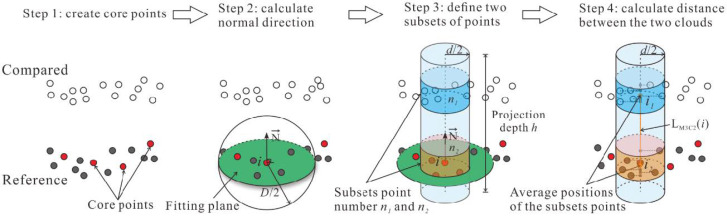
Cloud-to-cloud distance calculated by the M3C2 algorithm.

The principle of the M3C2 algorithm can be roughly described in four steps, as shown in Figure 7. First, the core points are selected in the reference model in Step 1. The normal direction of each core point is subsequently calculated by using a fitting plane controlled by a set of normal scales *D* in the software. Then, a cylinder along the known normal direction is created by setting the projection depth *h* and the projection scale *d* of the cylinder. The two subsets of points from two 3D models within the cylinder are detected in Step 3. Finally, the distance between the two subsets of points is obtained by calculating the length of the average position of each subset along the normal direction. More details about the M3C2 algorithm are described by Lague et al. [33].

Four settings, including the core points, the normal scale *D*, the projection scale *d*, and the projection depth *h* in the software, need to be defined by the user. Considering the spatially homogeneous points in the HLS model, all HLS points were chosen as the core points in the calculation. Sensitivity analysis was performed by using the GUESS function in CloudCompare to obtain the parameters *D*, *d*, and *h*. Here, the parameters *D*, *d*, and *h* were 0.573, 1.146, and 10.28 mm for all models, respectively.

#### 2.4.2. JRC Error

To make a 2D assessment of the SfM models, JRCs were used and calculated from the 2D profiles of the SfM and HLS models. In the 2D JRC calculation, the parameter Z_2_ is calculated as follows [34]:

(1) $$Z_{2}={\left[\frac{1}{M{\left(P\right)}^{2}}\sum_{i=1}^{M}{\left(y_{i+1}-y_{i}\right)}^{2}\right]}^{1/2},$$

where *M* is the number of intervals along the entire length *L*, *P* is the point interval, and *y_i_* is the height of the profile. The parameter *Z*_2_ has been widely used to characterize the roughness of rock joint surfaces, and many regression equations have been established between *Z*_2_ and JRC. Yu and Vayssade [35] proposed a set of regression equations between *Z*_2_ and JRC values with different uniform point intervals as follows:

(2) $$JRC=60.32Z_{2}-4.51\;(\mathrm{Point}\;\mathrm{interval}:\;0.25\;\mathrm{mm}),\;$$

(3) $$JRC=64.22Z_{2}-2.31\;(\mathrm{Point}\;\mathrm{interval}:\;1.0\;\mathrm{mm}),\;$$

which has been widely used in JRC estimation [36]. Since the point interval of the SfM model was nonuniform, according to Equations (2) and (3), the SfM models needed to be resampled with point intervals of 0.25 and 1.0 mm by using the MountainsMap software. The HLS models were also resampled with the same point interval for comparison. In this study, ten 2D profiles were extracted at even spacing from each resampled SfM and HLS model, and the profiles were named the SfM profiles and HLS profiles, respectively. Then, the JRC of each 2D profile was calculated by using Equations (1)–(3). Here, *JRC_SfM_* and *JRC_HLS_* represent the JRCs of the SfM and HLS profiles, respectively.

The *JRC_error_* is defined as

(4) $$JRC_{error}=\frac{JRC_{SfM}-JRC_{HLS}}{JRC_{HLS}}$$

The *JRC_error_* can indicate the accuracy of each SfM profile. Thus, the mean value of ten *JRC_error_*s was used to evaluate the accuracy of the SfM photogrammetric method in JRC estimation.

## 3. Results

### 3.1. Three-Dimensional Reconstruction of Rock Joints through SfM Photogrammetry

A total of 40 image datasets were collected for SfM reconstruction with different devices, rock joint specimens, and shooting strategies. In the 3D reconstruction process, three image datasets with a shooting angle of 30° were not aligned, causing the 3D reconstruction to fail. Finally, a total of 37 SfM models of rock joints were generated.

Table 4 and Table 5 show the average point density and nominal point interval of the SfM model using the different devices under the FSC mode and the MSC mode, respectively. The size of each SfM model is slightly different in the clipping process in the CloudCompare software. The number of points of each SfM model with different smartphones is quite different, ranging from 152,000 to 1,870,000. For the T329t, only approximately 500,000 points or less are in the SfM models.

**Figure 8:**
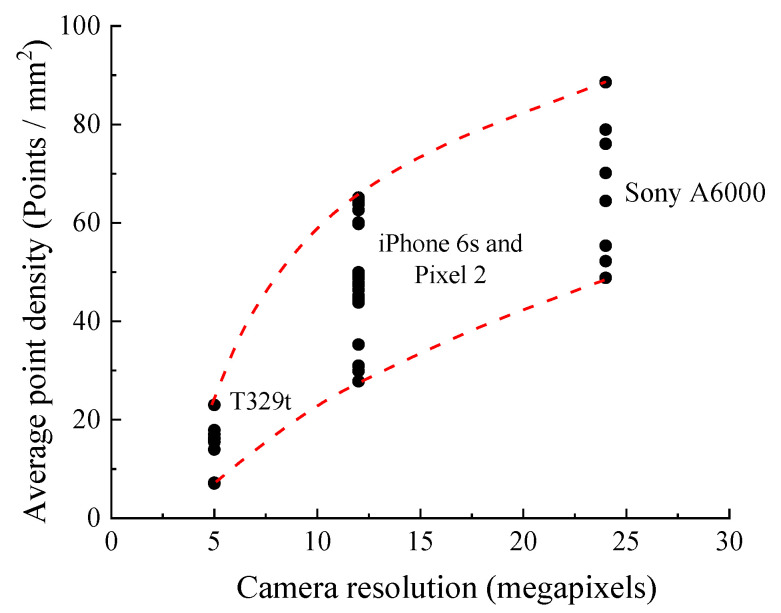
The average point density of each structure from motion (SfM) model that is generated by using the different cameras.

The average point density *ρ* and the nominal point interval *T* are calculated using the following equations:

(5) $$\rho =\frac{n}{s},$$

(6) $$T=\frac{1}{\sqrt{\rho }},$$

where *n* is the number of points and *s* is the size of the SfM model. The two values can reflect the effect of the camera quality on the SfM model. Note that all points of each SfM model are assumed to follow a uniform distribution. For the T329t with a 5 MP camera, the density *ρ* and interval *T* are 7–18 points/mm^2^ and 0.21–0.38 mm, respectively; in addition, both the iPhone 6s and Pixel 2 with 12 MP cameras generate the SfM model with 28–65 points/mm^2^ and 0.12–0.19 mm, respectively. In addition, the Sony A6000 high-end camera shows a larger density *ρ* and smaller interval *T* of 49–89 points/mm^2^ and 0.11–0.14 mm, respectively. Figure 8 shows the average point density of each SfM model with different camera resolutions. As the camera resolution increases, the density *ρ* of the SfM models increases nonlinearly.

### 3.2. Cloud-to-Cloud Distance

Table 6 and Table 7 show the cloud-to-cloud distance (M3C2 distance) between the SfM and HLS models. The 1st percentile and 99th percentile M3C2 distances are given. The results show that 98% of the absolute M3C2 distances are within 0.3 mm for each SfM model except the two cases by using T329t. In addition, the mean, standard deviation (SD), and root mean square error (RMSE) of the M3C2 distances were calculated. Regarding the standard deviation and RMSE, these two values are close, since the mean value is close to 0. Following the digital geospatial accuracy standards created by the American Society for Photogrammetry and Remote Sensing [37], the RMSEs of the M3C2 distance, also called the RMSEs of the SfM models, were used to represent the accuracy of the SfM models. The influence of the camera quality, specimen texture, and shooting strategies on the quality of SfM models are given as follows.

#### 3.2.1. Camera Quality and Specimen Texture

**Figure 9:**
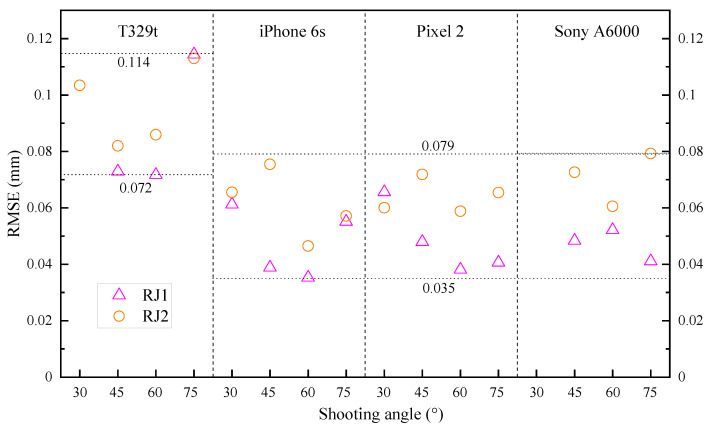
The root mean square error (RMSE) of the SfM models of RJ1 and RJ2 with the different cameras and different shooting angles under the FSC mode.

Figure 9 demonstrates the RMSE of the cloud-to-cloud distance through different devices for two rock joint specimens under the FSC mode. In general, all SfM models generated by the four cameras show good performance with low RMSEs, which range from 0.036 to 0.115 mm. Relatively poor results were obtained by using the T329t, with RMSEs of 0.072–0.115 mm. In contrast, the applications of the iPhone 6s and Pixel 2 with the same camera resolution have similar RMSEs of 0.036–0.080 mm. The performance of the Sony A6000 high-end camera does not significantly surpass that of the iPhone 6s and Pixel 2.

From Figure 9, the RMSEs of the RJ1 models are lower than those of the RJ2 models under the same conditions, except for the case with a shooting angle of 30° using the Pixel 2. Although the reconstruction quality of natural rock joint RJ1 is slightly better than that of artificial rock joint RJ2, the maximum difference in the RMSEs between the two specimens is only 0.038 mm. Therefore, it is inferred that the smartphone SfM photogrammetric method has acceptable performance for reconstructing rock joints with different textures.

#### 3.2.2. Shooting Strategy

**Figure 10:**
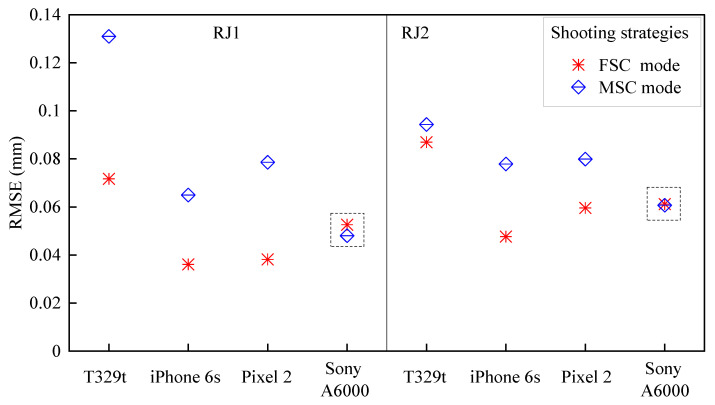
The RMSE of the SfM models under the MSC mode and FSC mode with a shooting angle of 60°.

Figure 10 shows the RMSEs of the SfM models under the MSC mode and FSC mode. Since the shooting angle under the MSC mode is between 50 and 70°, the SfM models with a shooting angle of 60° under the FSC mode are selected for comparing the effect of the shooting mode on the 3D reconstruction. For all SfM models using the three smartphones, the RMSE values under the MSC mode are smaller than those of the FSC mode with a maximum RMSE difference of 0.059 mm. For the SfM models using a high-end camera, the RMSE difference between the two capturing modes is tiny and less than 0.004 mm.

**Figure 11:**
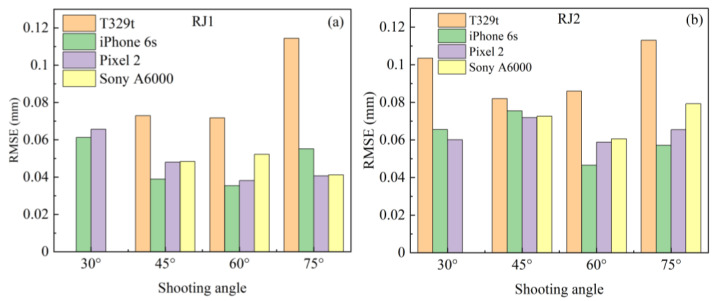
Comparison of the RMSE of the SfM models with the different shooting angles under the FSC mode, (**a**) specimen RJ1, (**b**) specimen RJ2.

Figure 11 shows the RMSEs of the cloud-to-cloud distance with the different shooting angles under the FSC mode. Note that three image datasets with a shooting angle of 30° failed to generate SfM models due to low image quality. For specimen RJ1, the SfM models with shooting angles of 45, 60, and 75° have similar performance except for that of the T329t with a shooting angle of 75°. For specimen RJ2, the SfM models obtained from the shooting angle of 60° always produce the lowest RMSE with a variation in the RMSE between 0.047 and 0.085 mm, except for the T329t SfM model. Therefore, the shooting angle of 60° under the FSC mode is recommended to collect image datasets.

### 3.3. JRC Error

**Figure 12:**
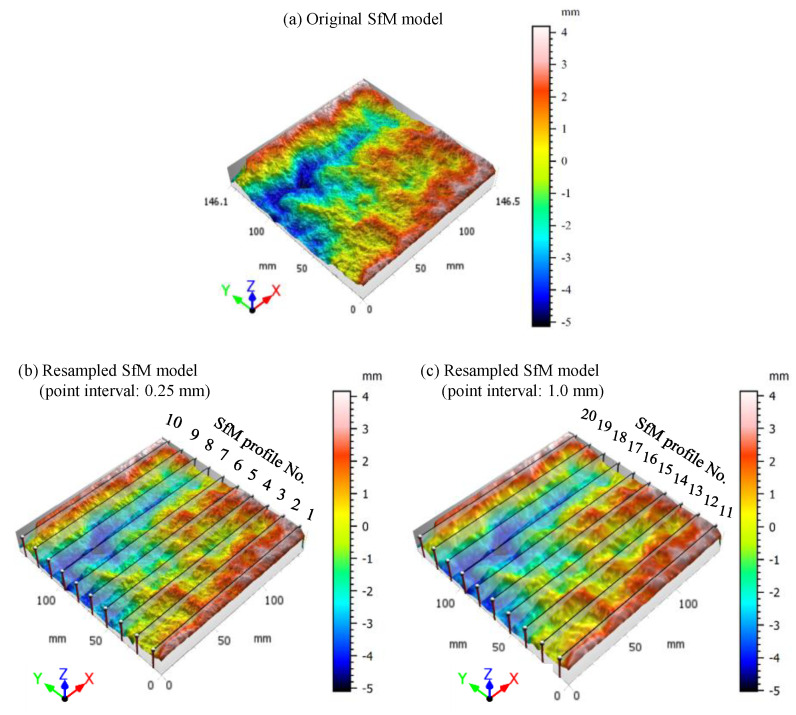
SfM models of RJ1 generated by Pixel 2 with a shooting angle 60°, (**a**) original SfM model, (**b**) and (**c**) resampled SfM models with point interval 0.25 and 1.0 mm, respectively.

Considering the excellent performance of the SfM model using the Pixel 2 with a 60° shooting angle, the model of RJ1 was applied to conduct the 2D JRC evaluation. Figure 12 shows the original SfM model and two resampled SfM models with point intervals of 0.25 and 1.0 mm, respectively. As the point interval increases, the resolution of the resampled SfM model gradually decreases. The 2D profiles no. 1–10 and profiles no. 11–20 are extracted from resampled SfM models with point intervals of 0.25 and 1.0 mm, respectively (see Figure 12). Additionally, twenty 2D profiles were extracted from the resampled HLS models at even spacing as a benchmark.

**Figure 13:**
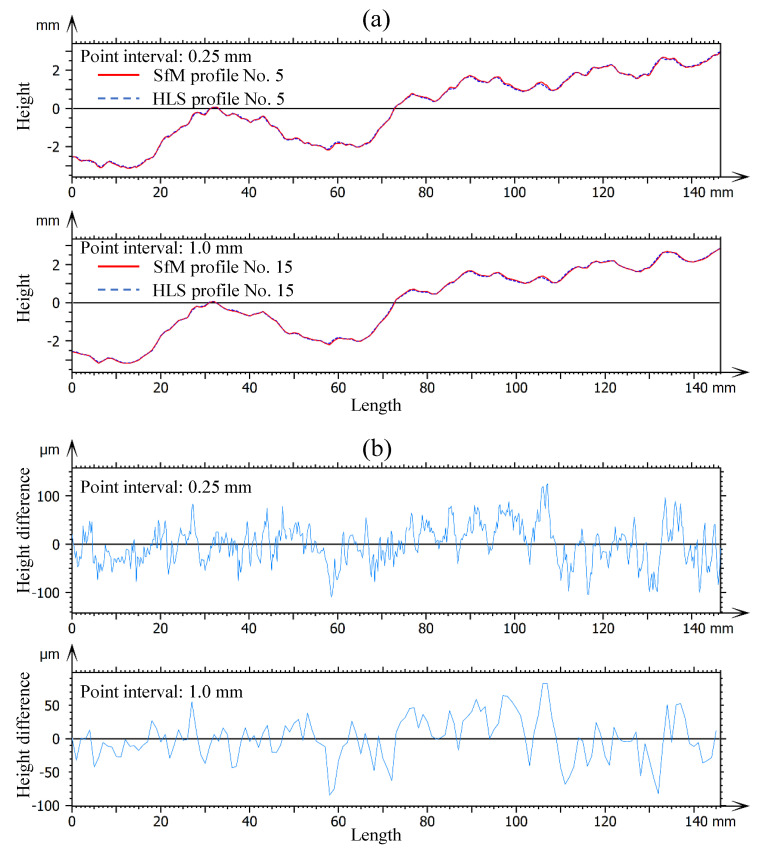
Comparison of the SfM and HLS profiles with different point intervals of 0.25 and 1.0 mm (**a**) from the height of profiles and (**b**) from the height difference of profiles.

Figure 13 shows two examples of the profile deviations of profile no. 5 and profile no. 15 with point intervals of 0.25 and 1.0 mm, respectively. The horizontal lines at a height of 0 mm represent the average height of the profile. Thus, the ordinate only represents the relative height of the profile. From Figure 13a, no visual difference can be found between the SfM and HLS profiles. For a detailed comparison, the height of the SfM profiles was subtracted from the height of the HLS profiles to obtain the height differences of the two profiles (see Figure 13b). The height differences with point intervals of 0.25 and 1.0 mm have a similar change along the horizontal length, but the line of the height differences with point intervals of 1.0 mm is smoother. The maximum height differences of the SfM and HLS profiles with point intervals of 0.25 and 1.0 mm are 120 and 80 μm, respectively. This indicates that as the point interval increases, the similarity of the SfM and HLS profiles slightly improves.

The JRCs of the profiles based on Equations (2) and (3) were calculated and summarized in Table 8. The *JRC_error_* of each SfM profile is also given. All the JRCs of the SfM profiles are less than those of the HLS profiles, which means that the SfM photogrammetry tends to underestimate the JRC of the rock joint surfaces. For profile no. 1–10 and profile no. 11–20, mean *JRC_error_* is −7.54 and −5.27%, respectively. This means that as the point interval increases, *JRC_error_* decreases.

## 4. Discussion

This section discusses the advantages and limitations of smartphone SfM photogrammetry for the measurement of laboratory-sized rock joint surfaces. Although most cases showed good performance, incorrect operations in image shooting or postprocessing in the software may decrease the accuracy or even cause the reconstruction of SfM models to fail. Therefore, some key factors affecting the accuracy of 3D models in the use of this method are also discussed below.

### 4.1. 3D Models

The accuracy of SfM models heavily depends on the quality of the image datasets. Generally, a high-quality camera and the correct imaging network configurations are necessary for an accurate 3D model. However, if the image resolution is too large (e.g., >12 MP), it is almost inevitable that the image needs to be resized to shorten the processing time [12]. In this study, the average point density of each SfM model through the Sony A6000 is 1.5–2 times the density of the models using the iPhone 6s and Pixel 2 and 4.5–8 times the density of the models using the low-end T329t smartphone. However, the RMSEs of the SfM models show that the performances of the iPhone 6s, Pixel 2, and Sony A6000 are close. This is different from the measurement results for the riverbank and alpine alluvial fan with a high-end camera and an iPhone 4 by Micheletti et al. [24]. This may be due to the 3D reconstruction in the laboratory-sized rock joint having a length of only 15 cm.

The reconstruction results of two specimens with different textures only have slight differences, which supports that smartphone SfM photogrammetry is capable of being applied for different types of rock joints. If the reconstruction quality is poor due to the textureless nature of a specimen, drawing or painting patterns on the surface of the sample is suggested to improve the quality of the 3D model. Furthermore, an advanced pattern projection technique can be adopted to improve the accuracy of the 3D model of textureless rock joints [38].

From the results of Section 3.2.2 for the shooting strategy, the MSC mode is an easy and effective shooting mode because no special preparation or equipment is needed, and the background in the pictures is beneficial for obtaining the camera position. Although the FSC mode requires careful preparation and may cause the failure of the model reconstruction due to improper operation, it can obtain 3D models with higher accuracy. Therefore, it is convenient and practical to choose the MSC mode for novices, while the FSC mode can obtain a more accurate SfM model for experienced users. This finding of higher accuracy in the FSC model is contrary to that of the conclusion proposed by Nikolov and Madsen [39]. They concluded that high accuracy of 3D models for six statues or vases was under the MSC mode. This discrepancy is likely related to the difference in equipment. A high-end camera (Canon 6D camera) with the good optical image stabilization technology can effectively reduce the effect of camera shaking in the MSC mode. However, images are easily out of focus or blurred in the MSC mode by using smartphones, which result in the lower accuracy of 3D models. In this study, the slight differences of RMSE are in the FSC and the MSC modes with Sony A6000 camera, which indicates that the two shooting modes by using the advanced camera have similar performance in the 3D reconstruction of rock joint surfaces.

**Figure 14:**
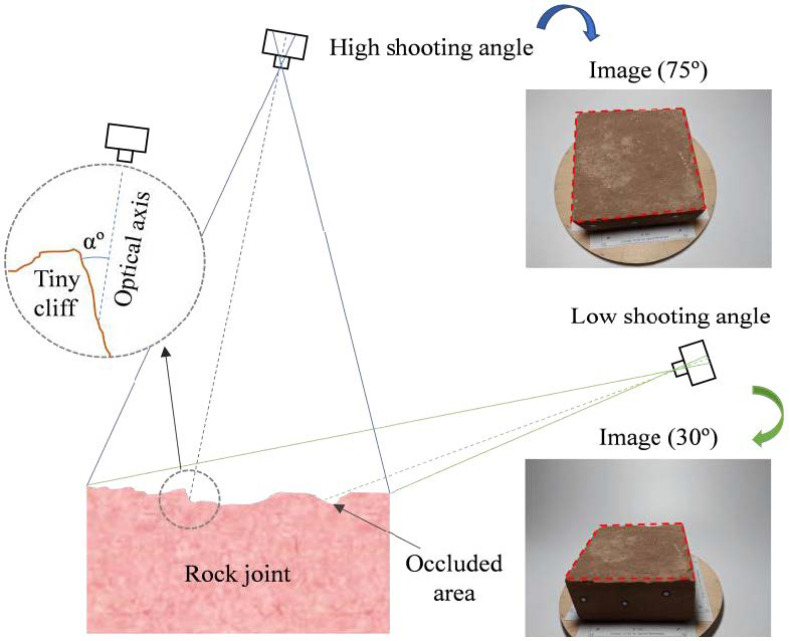
The influence of the camera shooting angle on the reconstruction of rock joint surfaces.

The optimum shooting angle mainly depended on the roughness of the rock joint surface, as shown in Figure 14. When the shooting angle is too low, the proportion of the sample surface in the frame is reduced, and local areas may be obscured. Similarly, when the shooting angle is too high, noisy points or voids may appear in the 3D models and construct tiny cliffs of the rock joint surface. The results of how the shooting angle affects 3D rock joint models agree with the results of the measurement of the rock surface using stereo photogrammetry by Kim et al. [40]. In general, shooting angles between 45 and 60° trade off capturing the details of the rock joint surface. Additionally, the overlapping photos obtained from various perspectives can effectively improve the accuracy of the 3D model, e.g., five sets of images with different intervals for statues or vases [39] and three rings of photos with different altitudes for spherical objects [41]. However, the larger numbers of images increase the workload and the processing time in the software.

### 4.2. Scale Bars

For the handheld sample, preparing scale bars for SfM photogrammetry is essential in the scaling of the 3D model because the size of the 3D model is random. Several solutions have been proposed, such as printing black dots on the turntable surface to simulate the scale bar [42] directly using a ruler [41] and using professional scale bars [43].

The accuracy of the SfM models is affected by the setup of the scale bars. In order to balance the accuracy and convenience, the scale bars were printed by using a laser printer HP LaserJet Pro MFP M126nw with a printing resolution of 600 × 600 dpi. The closest distance between two ink dots in the scale bars can reach 0.042 mm by the printer. We measured the scale bar by using a vernier caliper with an accuracy of 0.02 mm. The distance error of the two coded markers with a distance of 10 cm was less than 0.1 mm, indicating that the accuracy of the scale bars by using the laser printer can reach 1:1000.

**Figure 15:**
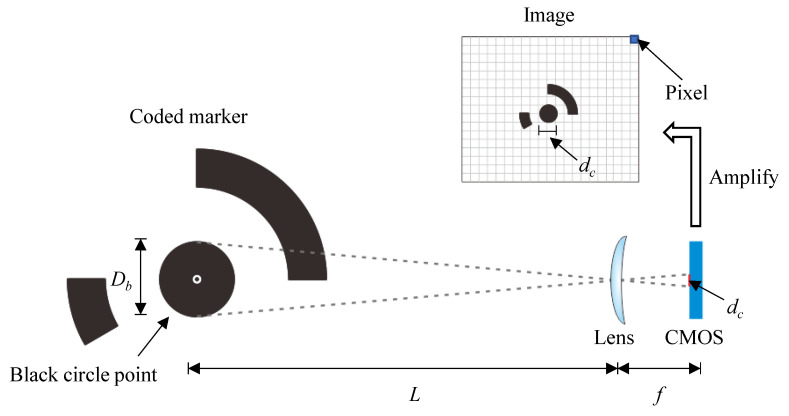
The basic principle of a smartphone camera.

To ensure that all the coded markers can be automatically detected in Metashape, the diameter of the black circle point *D_b_* in the coded markers needs to be limited to a specific size. Based on some preliminary checking, the diameter of the black circle point *D_b_* in the image is recommended to be less than 30 pixels. According to the basic principles of optical imaging (see Figure 15), the size of the coded marker on the COMS image sensor is proportional to the actual size of the coded marker as follows:

(7) $$\frac{D_{b}}{L}=\frac{d_{c}}{f},$$

where *D_b_* is the diameter of the center circle of the coded marker, *d_c_* is the actual size of the target imaged on the COMS sensor equal to the pixel size multiplied by the number of pixels, *f* is the focal length of the camera, and *L* is the distance from the camera to the object (camera to object). Here, *d_c_* equals the pixel size times the number of pixels. As for iPhone 6s, with the known focal length f of 4.15 mm, pixel size of 1.22 μm, and distance L of 30 cm, the diameter of the black circle point *D_b_* is suggested to be less than 0.264 mm.

### 4.3. Sources of Error

Identification of the sources of error in point cloud comparison is essential for evaluating the accuracy of SfM models. In the measurement of the rock joint surface, the errors of measurement results include photogrammetric error, registration error, and surface roughness related error. The details of these sources are as follows:

Photogrammetric error: the most critical error of the measurement result depends on the measurement tool itself. The sources include camera internal parameters (see Table 3), image capturing strategies (In Section 4.1), and data processing algorithm. Moreover, the accuracy of the scale bar is also an influencing factor for close range photogrammetry. The printed scale bar used in this study has been proven to have an accuracy of 1:1000.

Registration error: the coordinate system of the SfM model and HLS model have a systematic error, which is related to the applied method. In this study, the ICP algorithm, the most popular algorithm for accurate registration of 3D point cloud data, was used to register 3D point cloud data. In the processing, the threshold of root mean square difference was set to 1×10^-5^ m, which is much less than the result of cloud-to-cloud distance.

Surface roughness related error: the M3C2 algorithm, a variant point to point comparison method, was used for calculating the distance between the SfM model and the HLS model. In the processing, the normal scale *D* and the projection scale *d* were set to 0.573 and 1.146 mm, respectively. Thus, the local rough areas with a projection diameter of less than 1.146 mm were treated as flat surfaces, which affects the surface normal orientation for distance calculation [33].

### 4.4. Comparison with Current Solutions

The smartphone SfM photogrammetric method provides smaller sampling interval compared to contact profiling methods, such as profile comb and needle profilometers. Moreover, the measurement range of the contact profiling method is limited by the devices, while there is no limitation on smartphone SfM photogrammetry.

Noncontact profiling methods, such as shadow profilometry [44] and laser profilometry [45], have the characteristics of high speed and high accuracy. However, they are costly due to the small commercial market, and the complicated operations of the apparatus also discourage many researchers. In addition, they can only provide 2D profile information compared to smartphone SfM photogrammetry.

Three-dimensional laser scanners, considered the gold standard, produce 3D models of rock joint surfaces with a high accuracy of 0.03 mm. According to the presented results on the RMSE of the cloud-to-cloud distance less than 0.08 mm, the smartphone SfM photogrammetric method is able to offer a similar accuracy to the 3D laser scanner approach. However, the 3D laser scanners are too expensive for many research groups or individuals. In contrast, smartphone SfM photogrammetry can obtain 3D models with relatively high accuracy at an extremely low cost. In addition, the quick acquisition of image datasets and flexible workflow in SfM photogrammetry improve the efficiency of the 3D model reconstruction of rock joint surfaces. Furthermore, color information exists with SfM models, which can be further used for visual inspection and model display.

### 4.5. 3D Reconstruction of the Rock Joint Surfaces through a Smartphone

The high-quality models of the rock joint surface in this study were obtained from a combination of smartphone-supported data acquisition and computer-supported data processing. Implementing the whole workflow needs data transfer operation and cannot reconstruct the 3D models immediately.

Although the computational ability in a smartphone is limited, Muratov et al. (2016) demonstrated the 3D reconstruction of a high-quality model within one minute for whole processing using Samsung Galaxy Tab S2 [46]. For obtaining 3D models of rock joints in time, further investigation on cloud-based processing or a data auto processing system for 3D reconstruction totally relying on a smartphone is necessary for rock engineering, especially at site.

In addition, smartphone SfM photogrammetry will be tested in more conditions to determine its repeatability, and a *JRC_error_* model needs to be established using more tests to improve the JRC estimation accuracy. Additionally, a multi smartphone shooting setup is required to improve the efficiency of this method.

## 5. Conclusions

A smartphone SfM photogrammetric method for measuring laboratory-sized rock joint surfaces is proposed, and the accuracy of this method is evaluated. A total of 37 SfM models were obtained under different conditions, including three smartphones and one high-end camera, two specimens, and two shooting modes. A handheld laser scanner was used as a benchmark. Two indicators, the RMSE of the cloud-to-cloud distance and *JRC_error_*, were used to evaluate the accuracy of the SfM models. Based on the test results, the following conclusions are drawn.

1. The smartphone SfM photogrammetric method has comparable accuracy to 3D laser scanner approach for reconstructing laboratory-sized rock joint surfaces. Due to its high accuracy, high efficiency, and low costs, it is expected to become a popular method for measuring rock joint surfaces. In contrast to expensive 3D laser scanners, this method allows more researchers to have the opportunity to participate in the study of rock joint roughness.

2. An iPhone 6s or Pixel 2 equipped with a 12 MP camera has a similar performance to a high-end camera in 3D reconstruction with an RMSE less than 0.08 mm. However, for the T329t with a 5 MP camera, the RMSE is within 0.114 mm. Additionally, the specimen texture has only a slight influence on the accuracy of the 3D model.

3. The FSC mode is slightly better than the MSC mode, and the difference in the RMSEs between the two models is less than 0.059 mm. It is recommended that novices use the MSC mode, while skilled operators can use the FSC mode to obtain more accurate 3D models.

4. Smartphone SfM photogrammetry tends to underestimate the JRC of 2D profiles extracted from 3D models. As the point interval of the SfM model increases, *JRC_error_* decreases. The mean *JRC_error_*s are −7.54 and −5.27% for the SfM model with point intervals of 0.25 and 1.0 mm, respectively.

## Figures and Tables

**Table 1:** Basic parameters of three smartphone cameras and a mirrorless camera.

Camera	HTC T329t	Apple iPhone 6s	Google Pixel 2	Sony A6000
Sensor type	CMOS BSI *	CMOS **	CMOS	CMOS
Sensor size	1/2 inch	1/2.8 inch	1/2.6 inch	23.5 × 15.6 mm
Pixel size (μm)	--	1.22	1.4	3.92
Image resolution	2592 × 1552	4032 × 3024	4032 × 3024	6000 × 4000
Focal length (mm)	4	4.15	4.47	16–50
35 mm equivalent focal length (mm)	--	29	27	24–75
Aperture	f/2 (fixed)	f/2.2 (fixed)	f/1.8 (fixed)	f/3.5
Auto focus	YES	YES	YES	YES
Output format	JPG	JPEG	JPEG	RAW

* COMS BSI is illuminated complementary metal oxide semiconductor. ** CMOS stands for complementary metal oxide semiconductor.

**Table 2:** Specifications of the handheld 3D laser scanner (HLS) FreeScan X5.

Specification	FreeScan X5
Weight	0.95 kg
Dimensions	130 × 90 × 310 mm
Scanning area	300 × 250 mm
Single shot accuracy	0.030 mm
Volume precision	0.02 mm + 0.08 mm/m
Resolution	0.100 mm
Scan speed	350,000 scan/s
Scan range	100~8000 mm
Working distance	300 mm
Scan depth	250 mm
Light source	10-line laser ray

**Table 3:** The calibrated parameters of the smartphone cameras and the mirrorless camera with different image datasets.

Parameter	T329t	iPhone 6s	Pixel 2	Sony A6000
*f (pixels)*	2100.56 to 2189.46	3481.57 to 3552.56	3258.19 to 3294.53	6513.49 to 6593.53
*cx (pixels)*	−2.92 to 9.14	−7.77 to 0.69	4.23 to 14.97	−10.91 to 15.12
*cy (pixels)*	−17.58 to 27.47	−28.91 to 29.03	−24.36 to 12.53	−64.09 to 0.43
*k*1* (pixels)*	0.0447 to 0.0718	0.0278 to 0.0351	0.2526 to 0.3289	−0.0465 to 0.0038
*k*2 *(pixels)*	0.2943 to 0.7538	0.1969 to 0.3092	−2.1903 to −1.2479	0.4759 to 0.9692
*k*3 *(pixels)*	−3.6009 to −1.2472)	−1.1747 to −0.7467)	1.6688 to 4.6424	−9.0926 to −2.0190
*p*1 *(pixels)*	−1.1×10^−3^ to 1.1×10^−3^	−6.4 × 10^−4^ to −1.2 × 10^−4^	1.3×10^−4^ to 1.3×10^−3^	−3.1×10^−4^ to 1.1×10^−3^
*p*2 *(pixels)*	−3.4×10^−3^ to 5.7×10^−4^	−9.6 × 10^−4^ to −1.1 × 10^−4^	−8.5×10^−4^ to 2.1×10^−3^	−3.3×10^−3^ to −1.5×10^−3^

**Table 4:** The average point density and nominal point interval of the structure from motion (SfM) models generated with the different shooting angles under the FSC mode.

Specimen	Camera	Shooting Angle	Size		Point Count	Average Point Density (points/mm^2^)	Nominal Point Interval (mm)
			X (mm)	Y (mm)			
RJ1	T329t	30°	Not aligned				
		45°	145.3	145.3	359,971	17	0.19
		60°	146.0	146.0	297,249	14	0.21
		75°	146.1	145.2	342,453	16	0.20
	iPhone 6s	30°	145.6	145.2	1,377,051	65	0.11
		45°	145.8	145.8	1,276,795	60	0.11
		60°	145.9	145.5	1,269,231	60	0.11
		75°	146.5	144.4	980,992	46	0.13
	Pixel 2	30°	146.4	147.0	1,075,093	50	0.12
		45°	146.1	146.3	946,617	44	0.13
		60°	146.5	146.1	937,802	44	0.13
		75°	146.3	145.8	1,040,414	49	0.13
	Sony A6000	30°	Not aligned				
		45°	145.9	145.9	1,680,130	79	0.10
		60°	146.9	146.9	1,513,866	70	0.11
		75°	146.9	146.2	1,048,596	49	0.13
RJ2	T329t	30°	148.3	147.9	504,820	23	0.17
		45°	146.0	144.0	376,180	18	0.19
		60°	146.9	144.8	329,427	15	0.20
		75°	145.7	146.9	349,153	16	0.20
	iPhone 6s	30°	145.1	143.3	1,327,271	64	0.11
		45°	146.8	142.1	1,346,091	65	0.11
		60°	148.7	145.4	1,353,423	63	0.11
		75°	147.3	143.9	1,002,612	47	0.13
	Pixel 2	30°	146.5	142.7	999,420	48	0.13
		45°	146.8	142.4	1,028,325	49	0.12
		60°	146.3	143.3	946,858	45	0.13
		75°	146.5	144.3	1,041,779	49	0.12
	Sony A6000	30°	Not aligned				
		45°	142.6	144.4	1,565,978	76	0.10
		60°	144.6	144.0	1,342,247	64	0.11
		75°	147.1	143.6	1,870,939	89	0.10

**Table 5:** The average point density and nominal point interval of the SfM models under the moving smartphone capture (MSC) mode.

Specimen	Camera	Size		Point Count	Average Point Density (points/mm^2^)	Nominal Point Interval (mm)
		X (mm)	Y (mm)			
RJ1	T329t	145.3	144.6	151,924	7	0.27
	iPhone 6s	145.6	143.4	624,914	30	0.15
	Pixel 2	145.0	142.7	729,642	35	0.14
	SonyA6000	144.8	144.2	1,090,384	52	0.12
RJ2	T329t	144.9	140.9	144,105	7	0.27
	iPhone 6s	144.7	139.3	624,954	31	0.15
	Pixel 2	144.3	141.6	568,228	28	0.16
	SonyA6000	145.7	143.2	1,154,870	55	0.12

**Table 6:** The M3C2 distance between the HLS model and SfM model under the FSC mode.

Specimen	Camera	Shooting Angle	M3C2 Distance (mm)				RMSE (mm)
			1st Percentile	99th Percentile	Mean (mm)	SD (mm)	
RJ1	T329t	30°	--	--	--	--	--
		45°	−0.220	0.160	−0.005	0.073	0.073
		60°	−0.190	0.082	−0.004	0.072	0.072
		75°	−0.349 *	0.240	−0.006	0.114	0.115
	iPhone 6s	30°	−0.160	0.140	−0.003	0.061	0.061
		45°	−0.080	0.112	0.007	0.039	0.039
		60°	−0.065	0.097	0.008	0.035	0.036
		75°	−0.129	0.145	0.013	0.055	0.057
	Pixel 2	30°	−0.172	0.158	0.000	0.066	0.066
		45°	−0.137	0.112	0.001	0.048	0.048
		60°	−0.097	0.097	0.002	0.038	0.038
		75°	−0.125	0.076	−0.004	0.041	0.041
	Sony A6000	30°	--	--	--	--	--
		45°	−0.153	0.102	−0.005	0.048	0.049
		60°	−0.191	0.095	−0.007	0.052	0.053
		75°	−0.131	0.074	−0.004	0.041	0.041
RJ2	T329t	30°	−0.298	0.190	−0.021	0.103	0.106
		45°	−0.181	0.168	−0.008	0.082	0.083
		60°	−0.190	0.193	−0.013	0.086	0.087
		75°	−0.262	0.259	0.015	0.113	0.114
	iPhone 6s	30°	−0.139	0.128	0.005	0.066	0.066
		45°	−0.187	0.126	−0.002	0.075	0.076
		60°	−0.093	0.064	−0.009	0.047	0.048
		75°	−0.146	0.103	−0.003	0.057	0.057
	Pixel 2	30°	−0.085	0.125	0.015	0.060	0.062
		45°	−0.133	0.140	0.001	0.072	0.072
		60°	−0.147	0.090	−0.009	0.059	0.060
		75°	−0.165	0.163	−0.003	0.065	0.066
	Sony A6000	30°	--	--	--	--	--
		45°	−0.160	0.143	−0.009	0.073	0.073
		60°	−0.139	0.100	−0.008	0.061	0.061
		75°	−0.149	0.240	0.006	0.079	0.080

* The absolute M3C2 distances in the case is larger than 0.3 mm.

**Table 7:** The M3C2 distances between HLS model and SfM model under the MSC mode.

Specimen	Camera	M3C2 Distance (mm)				RMSE (mm)
		1st Percentile	99th Percentile	Mean (mm)	SD (mm)	
RJ1	T329t	−0.327 *	0.256	−0.017	0.131	0.132
	iPhone 6s	−0.153	0.154	0.004	0.065	0.065
	Pixel 2	−0.164	0.185	0.013	0.079	0.080
	Sony A6000	−0.115	0.122	0.002	0.048	0.048
RJ2	T329t	−0.200	0.214	−0.004	0.094	0.095
	iPhone 6s	−0.160	0.193	−0.007	0.078	0.079
	Pixel 2	−0.171	0.154	−0.001	0.080	0.080
	Sony A6000	−0.132	0.102	−0.004	0.061	0.061

* The absolute M3C2 distances in the case is larger than 0.3 mm.

**Table 8:** *JRC_error_* of the 2D profiles.

Profile No.	Point Interval: 0.25 mm			Profile No.	Point Interval: 1.0 mm		
	*JRC_SfM_*	*JRC_HLS_*	*JRC_error_*		*JRC_SfM_*	*JRC_HLS_*	*JRC_error_*
1	4.38	4.89	−10.37%	11	5.52	5.96	−7.23%
2	5.08	5.56	−8.67%	12	6.44	6.94	−7.31%
3	6.84	7.34	−6.74%	13	8.04	8.67	−7.26%
4	6.78	7.26	−6.64%	14	7.96	8.43	−5.63%
5	6.24	6.59	−5.40%	15	7.32	7.64	−4.12%
6	7.10	7.78	−8.76%	16	8.49	8.83	−3.93%
7	6.92	7.58	−8.75%	17	8.22	8.74	−5.95%
8	6.40	6.79	−5.69%	18	7.67	8.11	−5.39%
9	7.33	7.84	−6.46%	19	8.45	8.70	−2.81%
10	5.21	5.66	−7.89%	20	6.32	6.52	−3.05%
Mean *JRC_error_*			−7.54%	Mean *JRC_error_*			−5.27%

## Data Availability

Data sharing not applicable.
